# Ultrasonic Sound Guide System with Eyeglass Device for the Visually Impaired

**DOI:** 10.3390/s22083077

**Published:** 2022-04-17

**Authors:** Kevin Kim, Saea Kim, Anthony Choi

**Affiliations:** Department of Electrical & Computer Engineering, Mercer University, 1501 Mercer University Drive, Macon, GA 31207, USA; kevin.s.kim@live.mercer.edu (K.K.); saea.kim@live.mercer.edu (S.K.)

**Keywords:** sound localization, the visually impaired, ultrasonic sound, binaural localization, eyeglasses, modulation, demodulation, personal navigation, near field

## Abstract

The ultrasonic sound guide system presents the audio broadcasting system based on the inaudible ultrasonic sound to assist the indoor and outdoor navigation of the visually impaired. The transmitters are placed at the point of interest to propagate the frequency modulated voice signal in ultrasonic sound range. The dual channel receiver device is carried by the visually impaired person in the form of eyeglasses to receive the ultrasonic sound for the voice signal via demodulation. Since the ultrasonic sound demonstrates the acoustic properties, the velocity, directivity, attenuation, and superposition of ultrasonic sound provide the acoustic clue to the user for localizing the multiple transmitter positions by binaural localization capability. The visually impaired hear the designated voice signal and follow the signal attributions to arrive at the specific location. Due to the low microphone gain from side addressing, the time delay between the receiver channels demonstrates the high variance and high bias in end directions. However, the perception experiment shows the further prediction accuracy in end directions as compared to the center direction outcomes. The overall evaluations show the precise directional prediction for narrow- and wide-angle situations. The ultrasonic sound guide system is a useful device to localize places in the near field without touching braille.

## 1. Introduction

The visually impaired (someone who is blind or has uncorrectable eyesight to a near normal level, also named “the user” in this article) experience various difficulties in navigating their way through situations. Conventional methods such as service dogs, walking canes, tactile braille, audio alarms, etc. improve the safety and efficiency of navigation. Recently, smart devices based on the global positioning system (GPS), cameras, and sensors provide the haptic and/or audio information to the visually impaired to deliver the context around them [1,2,3,4,5]. However, navigation is still challenging in the final phase due to the position ambiguity of the destination [6]. With the lack of visual information, the visually impaired cannot specify an accurate destination position unless they have a priori experience. Indoor navigation increases the destination ambiguity further because of the structure similarity between locations. The smart devices may find the pinpoint location by using the cameras and sensors; however, the information delivery to the user is still a problem being confronted [7,8]. Note that smart devices require implementation complexity with inconsistent performance in general.

The simple solution to the challenges of the final phase navigation is that someone speaks the location name on the destination. The sound localization capability of humans allows them to find the destination location by nature. The direction and distance to the destination are speculated by the arrival signal difference between ears based on the magnitude, time, and frequency [9]. The audio speaker with a pre-recorded message or signal may provide the solution to the final phase navigation. Audio-based assistive devices are realized and deployed on the streets and sidewalks, especially at intersections [10]. The signal pole location and pedestrian crossing direction are guided by designated audio signals. The audio devices can be used for sports activities [11], point of interest identification [12,13], safe navigation [14], etc. of the visually impaired to support outdoor movements as well. The balance between accessibility and environment is required to maintain the sustainability of the assistive devices based on the audio signal. Because of the audible sound broadcasting, audio-based devices demonstrate the limited installation locations [15].

Humans demonstrate the accurate and extensive capability to localize sound sources on the horizontal and median plane with two ears [16]. The time and magnitude difference between ears allow the listener to estimate multiple sound locations on the horizontal plane. The spectral variation by the asymmetric pinna structure provides the median plane localization. By combining the differences and variation along with the head related transfer function, the human perceives the delicate information from the received acoustic sound for precise localization on three-dimensional (3D) space. Various sound localization systems [17,18] have been proposed to imitate the human auditory system. In recent times, scientists have completed studies to understand the propagation on the practical architecture with dedicated structure for precise and feasible sound source localization systems [19,20,21,22,23].

In academia, a navigation system designed for the visually impaired has been a challenging topic because of the situation complexity and the human–machine interface. For decades, numerous studies have been published to solve the various aspects of navigation. Extensive review articles [24,25,26,27,28] have been proposed to understand and promote the latest technologies for navigation. El-taher et al. [28] present a comprehensive review of research relevant to the assistive outdoor navigation with a series of phases and tasks. Kuriakose et al. [27] consider major solutions that work in both indoor or/and outdoor environments based on various technologies. In addition to the extensive reviews, Simões et al. [26] discuss a series of considerations and future trends for the construction of indoor navigation and location systems. With relevance to this study, Bai et al. [29] suggest smart guiding eyeglasses based on depth and ultrasonic sensors to detect obstacles. The ultrasonic sound localization system presented by Pulkki et al. [30] is designed by the microphone array with a spatial synthesis process to monitor wildlife and man-made systems.

Beyond the academic approaches, the practical methods for the navigation system have been proposed as a patent. The wearable computing system maintains the safe linear and turn path for the visually impaired by using the haptic vibration and/or audio speaker [31]. The portable electronic device guides the visually impaired based on wireless communication [32], light communication [33], or a beacon speaker [34] from location transmitters or an information terminal. The intelligent pedestrian access system provides safe navigation on the pedestrian crossing [35]. The assisted walking device presents audible directions for the desired destination based on GPS [36]. The eyeglass-based device delivers tactile feedback to maintain eye contact with a speaking person based on the audible sound difference between two microphones [37].

The inaudible sound propagation with a proper receiver device overcomes the installation problems since the information is delivered to the selective persons who possess the receiver device. The ultrasonic sound presents the frequency band beyond the audible range with acoustic properties which are the velocity, directivity, attenuation, and superposition of the signal [38,39]. Hence, the audio signal carried by ultrasonic sound delivers the identical localization clues to the receiver device user by following the signal strength and delay. In order to improve the usability, the receiver device is realized in the wearable form as eyeglasses. The dual channel configuration in the eyeglass device improves the final phase navigation performance further by employing the human binaural localization capability explicitly. The channel indicates the individual receiver processing path from the single microphone to the single speaker. The dual channel device independently receives the ultrasonic sound from two microphones which have the physical distance in between. The ear with the individual speaker hears the sound from the channel with time and magnitude difference for human binaural sound localization. In addition, it is important that the visually impaired maintain the ambient sound localization capability; hence, the receiver device must be configured as open structure to keep the external acoustic flow going to the ear canal.

**Figure 1:**
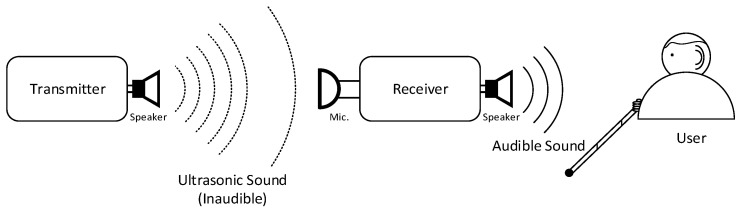
Schematic block diagram of the ultrasonic sound guide system for the visually impaired.

The ultrasonic sound guide (USG) system provides the proximity localization system and method for the visually impaired to improve their personal navigation performance in a near destination situation. As shown in Figure 1, the system consists of a transmitter and receiver device to broadcast and receive the ultrasonic sound, respectively. The transmitter is placed on the destination to deliver the specific audio signal to indicate the location information. The transmitter shifts the audible frequency to the ultrasonic frequency by using frequency shift; hence, the naked ear cannot hear the information. The receiver device is carried by the visually impaired person (shown as user in Figure 1) in order for them to find the transmitter location by following the signal attributions. The receiver in the device performs the demodulation by shifting the ultrasonic frequency to the audible frequency. The physical movement of the receiver device is required, such as horizontal and vertical rotation, in order to discover the transmitter direction. Once the visually impaired person observes the direction by the movement, the user progresses to the direction and experiences the stronger and consistent audio from the receiver device. The direction and range between the transmitter and receiver device determine the audio signal delay and attenuation factor, similar to the acoustic sound characteristics.

## 2. Methods

The USG system provides the acoustic information to the visually impaired person to identify and localize the sound source based on the ultrasonic sound. The sound source indicates the ultrasonic sound transmitter which is placed on the purposed destination in the personal navigation. The ultrasonic sound is generated by the frequency shift on the voice spectrum to deliver the place name continuously and selectively. The receiver device carried by the visually impaired person accepts the ultrasonic sound and shifts back into an audible frequency to identify the location. In addition, the audible sound strength by the receiver device direction and distance presents the clue to finding the transmitter location. Two receivers with physical distance between microphones can be configured as the dual channel device for further localization capability. The two speaker outputs from the dual channel device are delivered to the individual ears directly. The arrival sound difference in magnitude and time provides the enhanced sound localization performance based on the binaural sound localization by the human. The USG system reduces the installation limitation since the information is selectively delivered to the receiver device by utilizing the inaudible ultrasonic sound.

### 2.1. Receiver Device in the Shape of Eyeglasses

**Figure 2:**
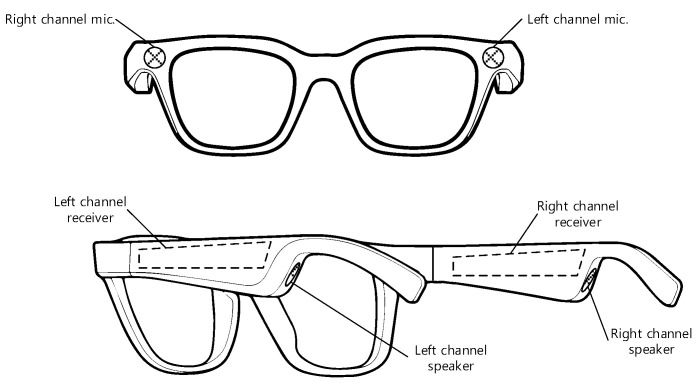
Receiver device in the shape of eyeglasses.

The physical structure of the transmitter has a high degree of freedom as long as the speaker propagates the ultrasonic sound properly. The physical shape of the receiver device requires careful design in order to maintain the surrounding spatial acoustic information of the visually impaired. Figure 2 demonstrates the receiver device, which is realized as dual channel configuration for the shape of eyeglasses. The individual temple of the eyeglass receiver device contains each channel receiver in symmetric manner. Individual channel comprises the receiver hardware, microphone, and speaker for independent processing. Two microphones are located at the left-end and right-end of rim front in the eyeglass receiver device. The distance between the microphones in dual channel device is approximately equivalent to the ear distance of the user. Two speakers are located at the back end of the temples near the user’s ears. The speaker outputs of the dual channel are directly provided to the individual ears in open structure. The power switch and battery system should be placed at cautious locations to consider the accessibility and safety of the user. The dual channel device presents the spatial acoustic information of the transmitter and maintains the environmental sound over the user. The numerous transmitters can be localized simultaneously by the dual channel device because of the conventional superposition of acoustic signals. The user can hear the multiple sounds from the receiver device and follows the desired sound strength and delay for specific transmitter location.

**Figure 3:**
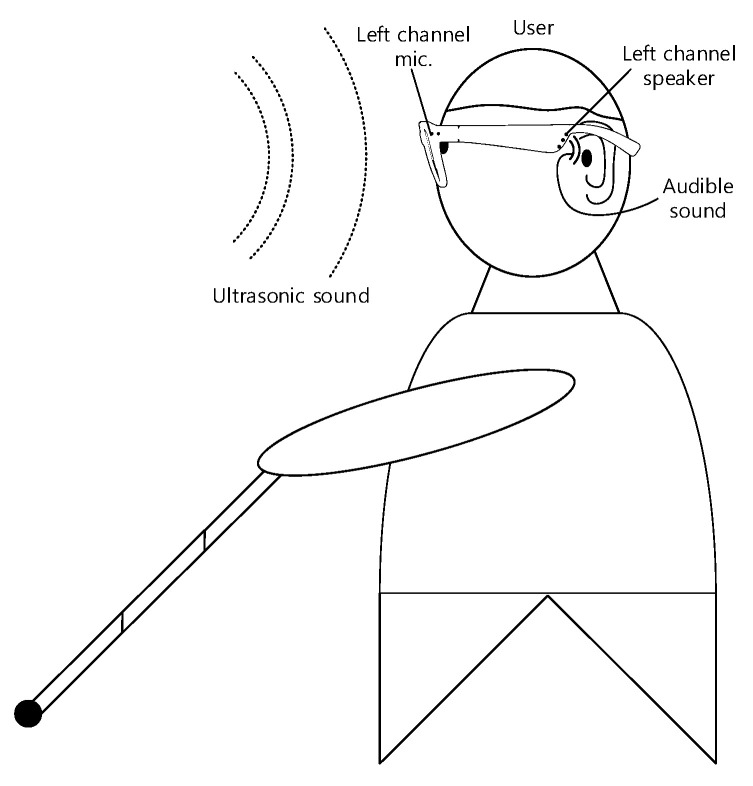
Example of an individual wearing the eyeglass receiver device.

Figure 3 illustrates an example of an individual wearing the eyeglass receiver device. The visually impaired person wears the eyeglass receiver device and holds the walking cane in one hand. Note that one side of the eyeglass receiver device is shown in Figure 3; the other side is symmetrical and identical. The two microphones of the eyeglass receiver device accept the ultrasonic sound from the transmitter. The receiver system converts the ultrasonic sound to the audible sound emitted by receiver speakers. Based on the dual channel device, the method to localize and approach the transmitter will be explained and illustrated in Section 2.4.

The USG system with eyeglass receiver device presents the transmitter localization by providing the acoustic clues to the user. The binaural capability of the user estimates the transmitter locations based on the sound difference between channels. Therefore, the USG system delivers the limited functions in the comprehensive and complex navigation of the visually impaired person. In particular, the USG system belongs to the phase of destination identification and localization at a close range. Along with the USG system, the device or method to avoid the obstacles such as walking cane and/or service dog is mandatorily required for the safe and optimized navigation, as shown in Figure 3.

### 2.2. Transmitter

**Figure 4:**
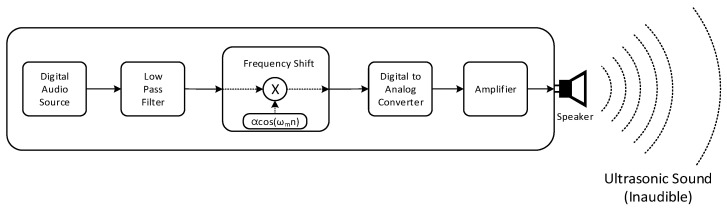
Simplified schematic block diagram of the transmitter.

The transmitter shown in Figure 4 delivers the audio information to the receiver by ultrasonic sound. The audio information contains the acoustic signal to identify the places or locations, such as room number. The analog audio signal is sampled and quantized by analog-to-digital converter (ADC) with $f_{s}$ rate (samples per second) and B bit resolution (bits per sample). The digitized audio signal is stored in the memory as digital audio source in Figure 4. In every $1/f_{s}$ time interval, the digitized audio signal is released from the digital audio source to the next processing block. The digitized audio signal is processed by the low pass filter (LPF) to limit the bandwidth of the audio signal. The filtered audio signal is shifted to the desired frequency range by using the sinusoidal signal, for instance $\alpha \cos\left(\omega_{m}n\right)$. The α is the magnitude control coefficient and the $\omega_{m}$ is the shift amount parameter. Observe that the time in digital signal processing is represented by integer sequence number n and the actual time is specified as $n/f_{s}$. The radian frequency ω represents the frequency from the discrete time domain n with radian per sample unit.

In frequency shift block, the time domain multiplication (denoted by x in Figure 4) between the sinusoid and filtered audio signal performs the frequency shift for desired frequency relocation. The shifted audio signal is converted to the analog signal by using the digital-to-analog converter (DAC). The analog signal is amplified by the amplifier. The amplified signal is transformed into the ultrasonic sound by using the speaker. The operation frequency range of the amplifier and speaker should be extended to the desired ultrasonic bandwidth for accurate propagation of the ultrasonic sound. Note that the frequency shift without the LPF may generate the unwanted high pitch audible sound to the user.

**Figure 5:**
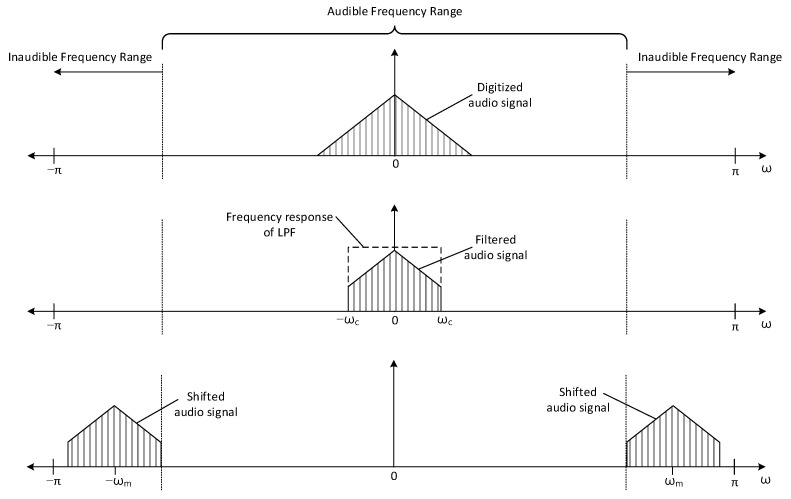
Frequency distribution of the individual transmitter block.

Figure 5 illustrates a frequency distribution of an individual transmitter block. The x axis of each plot represents the radian frequency ω and the y axis of each plot specifies absolute signal magnitude of the given frequency. Due to the property of discrete Fourier transform, the frequency distribution demonstrates the even symmetric profile on absolute magnitude. Note that the upper audible frequency limit is 20 kHz in general. The individual plots in Figure 5 present the range of audible and inaudible frequency. The triangular shape in the upper plot of Figure 5 shows the frequency distribution of digitized audio signal in digital audio source in Figure 4. The pentagon shape in the middle plot of Figure 5 demonstrates the frequency distribution of filtered audio signal. The rectangular dotted line in the middle plot of Figure 5 provides the frequency response of Figure 4 LPF, which passes the signal up to $\omega_{c}$ frequency. Two shifted pentagon shapes in the bottom plot of Figure 5 denote the frequency distribution of shifted audio signal. The frequency shift in Figure 4 performs the frequency relocation in right and left direction with shift amount $\omega_{m}$. Observe that the shifted audio signal occupies the inaudible frequency range in the bottom plot of Figure 5; therefore, naked ears cannot hear any audible sound from the transmitter in Figure 4.

### 2.3. Receiver

**Figure 6:**
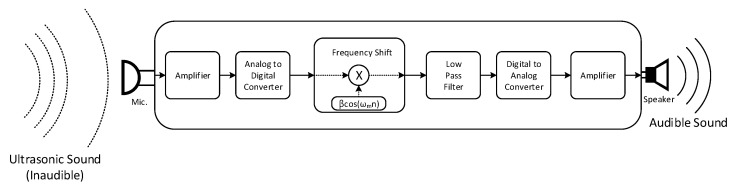
Simplified schematic block diagram of the receiver.

The receiver demonstrated in Figure 6 receives ultrasonic sound which contains the audio information and converts it to the audible sound for the visually impaired person. The microphone accepts the wide-band sound and the amplifier magnifies the received analog signal for further processing. The analog signal is sampled and quantized in real time by ADC with $f_{s}$ rate and B bit resolution. The digitized signal is shifted to the desired frequency range by using the sinusoidal signal, for instance $\beta \cos\left(\omega_{m}n\right)$, in frequency shift. The β is the magnitude control coefficient and the $\omega_{m}$ is the shift amount parameter. In frequency shift block, the time domain multiplication between the sinusoid and the signal performs the frequency shift for desired frequency relocation. The LPF passes the desired frequency signal and blocks the high frequency component in order to recover the propagated audio signal. The filtered audio signal is converted to the analog signal by using the DAC. The analog signal is amplified by the amplifier. The amplified signal is transformed into the audible sound by using the speaker. The receiver should be responded to rapidly for user safety; hence, all operations are performed in real time to minimize the latency.

**Figure 7:**
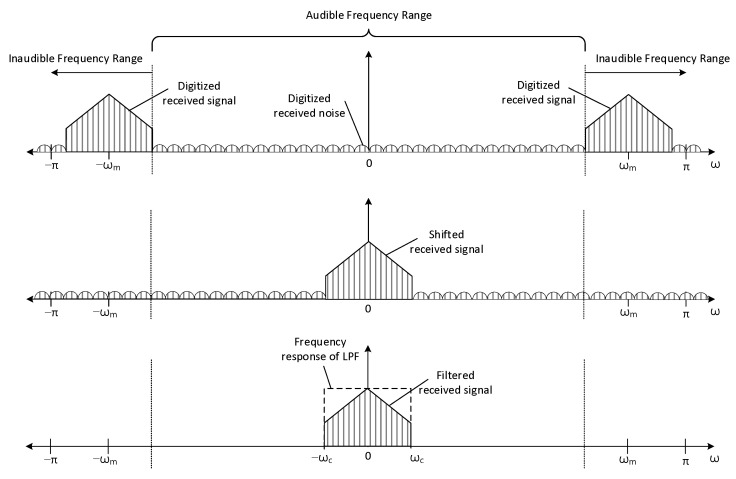
Frequency distribution of the individual receiver block.

Figure 7 presents the frequency distribution of the individual receiver block. The x axis represents the radian frequency ω and the y axis specifies absolute signal magnitude of the given frequency. The upper plot of Figure 7 demonstrates the frequency distribution of the digitized received signal after ADC on Figure 6. The pentagon shapes in the upper plot of Figure 7 are induced from the propagated transmitter signal and occupy the inaudible frequency range. The ripples are wideband received noise from the ambient sound and microphone. The frequency shift performs the frequency relocation in right and left direction with shift amount $\omega_{m}$. In the middle plot of Figure 7, one of the shift directions is arranged and superimposed around the zero frequency. The bottom plot of Figure 7 illustrates that the received and shifted noise is removed by using the LPF, which passes the signal up to $\omega_{c}$ frequency. The dotted lines in the bottom plot of Figure 7 provide the frequency response of Figure 6 LPF. The clean shifted signal is placed in the audible frequency range; hence, the visually impaired person can hear the audible information from the receiver.

### 2.4. Localization

The proposed eyeglass receiver device is configured as a dual channel device which represents the receiver device with two independent processing channels from two microphones to two speakers. On an individual microphone, each channel performs the receiver operations shown in Figure 6 to deliver the audible information by using the speaker. The human operates the binaural sound localization in 3D space by accepting the two-ear sound receptions with the pinna around the canal. Over the human head, the horizontal plane presents the binaural sound localization environment due to the ear positions. The unique pinna structure provides the acoustic variation of multiple reflections to identify the arrival angle over the median plane. The eyeglass receiver device is designed to find the transmitter directions in horizontal plane; hence, the distance between the microphones is approximately equivalent to the distance between the ears. The receiver speakers are placed near the ears with open structure in order to hear the sound difference between channels in magnitude and delay. Note that the acoustic flow of the ear should not be blocked by the receiver system to maintain the spatial acoustic sensing of the visually impaired person.

**Figure 8:**
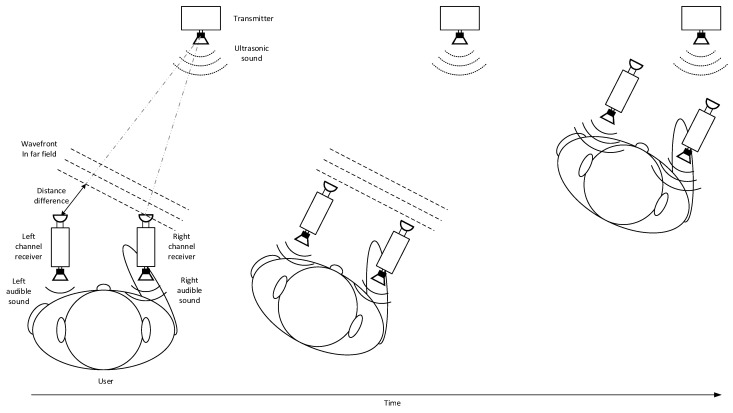
Working example of dual channel device for user to locate the transmitter.

Figure 8 demonstrates the sound guide processing by the user based on the dual channel device. Note that the illustration is depicted by the dual channel device for improved explanation without the actual eyeglass receiver device figure due to the identical working process. Three pictures are shown horizontally in temporal order from left to right. The left, middle, and right picture contain the identical configuration as below. The transmitter broadcasts the ultrasonic sound by using the speaker. The ultrasonic sound is propagated into the air and the wavefront of the ultrasonic sound exhibits the plane wave in far field. The user possesses the dual channel device in parallel to ear line similar to the eyeglass receiver device. The dual channel device includes two microphones, two receivers, and two speakers in symmetric fashion. The receiver speakers are located near the individual ear of the user. In the left picture, the user is positioned at the far location with indirect direction and receives the audible sound from the transmitter for location identification. The user experiences the audible sound difference between the receiver speakers due to the direction of dual channel device. The direct distance difference between microphones creates the audible sound difference in magnitude and delay. The human binaural sound localization capability perceives the direction of transmitter and the user turns the body direction toward the transmitter in the middle picture. The user in the middle picture faces toward the transmitter and experiences the even audible sound in level and delay from the receiver speakers. The user in the right picture approaches the transmitter for higher audible sound level and understands that the user arrived at the destination.

## 3. Experiments

The USG system realization requires extensive real-time processing to transmit and receive the desired signals. The USG system in this study is implemented by the digital technology based on the processor and ADC/DAC. The tight time budget of realization prevents the distortion of the audio and localization information, which ensures the safety of the visually impaired. In terms of the physical profile, the transmitter has a high degree of freedom of shape as long as the speaker is placed under the acoustic hole for propagation. The receiver device is configured with dual channel structure as the eyeglasses; therefore, the user should wear the receiver device which includes all necessary parts inside. To maintain the wearability, the eyeglass receiver device in this experiment is embodied with wireless technology and conventional off-the-shelf products. Observe that the receiver process shown in Figure 6 is realized as the embedded programming in the system processor.

The experiments in this article are divided into two categories as the perceptual and acoustic investigation. The perceptual experiment measures the horizontal localization performance by blindfolded human. The person who wears the eyeglass receiver device is requested to find the direction of transmitter in various conditions. The acoustic experiment evaluates the delay of the received signals in the eyeglass device. Note that the delay indicates the time difference for inter-microphones’ configuration in the receiver device. The experiments are performed at an indoor location which presents the deteriorated environments due to the echoes. The USG system is expected to function better in anechoic locations since indirect paths are reduced by open and/or absorptive structure.

### 3.1. Realizations

This section provides the actual realization of the USG system comprised of the transmitter and receiver. The transmitter generates the predefined message continuously; therefore, the signal is preprocessed and stored in the computer after the LPF and frequency shift stage shown in Figure 4. The playback by computer program releases the ultrasonic sound via audio interface and speaker based on the DAC and amplifier process. The receiver device in eyeglasses is implemented by two independent embedded processors for dual channel configuration. The processor board includes all required receiver hardware except the receiver speakers. The audio eyeglasses from off-the-shelf product are employed as the device form factor with the receiver speakers.

#### 3.1.1. Receiver Device

**Figure 9:**
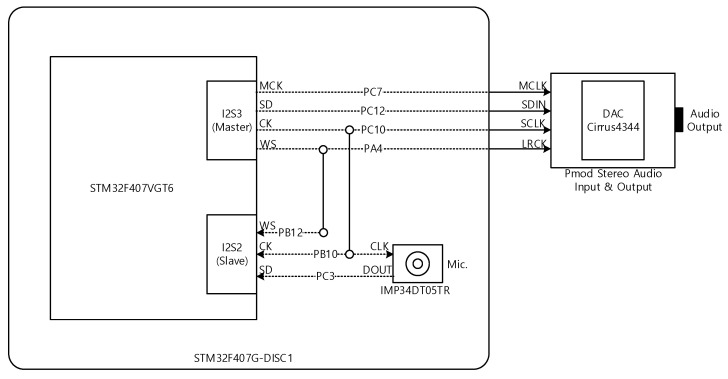
Hardware configuration of individual channel receiver. (SD specifies the serial data).

The receiver device requires significantly low and consistent latency for receiver processing due to the time sensitive information between the channels. In order to satisfy the requirement, the individual channel is processed by the dedicated processor (STM32F407VGT6, STMicroelectronics, Geneva, Switzerland) which includes the 32-bit Arm Cortex M4 architecture with floating-point unit (FPU). The processor and peripheral devices are placed on the development board (STM32F407G-DISC1, STMicroelectronics, Geneva, Switzerland) for integrated programming environment. The peripheral devices contain the omni-directional digital microphone (IMP34DT05TR, STMicroelectronics, Geneva, Switzerland) for receiving the wide-band sound. The external audio input and output device (Pmod I2S2, Digilent, Pullman, WA, USA) is connected to the development board by using the Inter-IC Sound (I^2^S) bus for DAC processing. The processor provides two I^2^S buses as I2S2 and I2S3 which are associated with the digital microphone and DAC processing, respectively. The I2S3 is served as the master for delivering the master (MCK), serial (CK), and word select (WS) clock and the I2S2 is operated as the slave for accepting the CK and WS clock. The overall hardware configuration of individual channel is demonstrated in Figure 9. Note that the PA4 indicates the Port A bit number; 4 pin and 9 ports (Port A~Port I) are available in STM32F40xxx processor.

**Figure 10:**
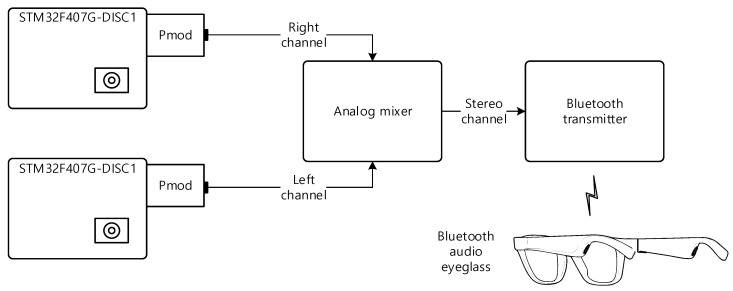
Eyeglass receiver device realization.

The individual channel receivers should be combined together as the left and right channel for eyeglass receiver device. To maintain the user accessibility, the Bluetooth audio eyeglasses (Bose Frames Alto, Bose Corporation, Framingham, MA, USA) is utilized for receiver form factor and the audio eyeglasses provides the stereo sound via speakers located at the end of each temple. The channel receivers are separated from the audio eyeglasses by using the Bluetooth connection; hence, the user experiences the actual prototype without any wires on the device. Due to the separation, observe that the user motion does not correspond with the receiver device movement. Figure 10 represents the overall system connections for the eyeglass receiver device. The analog mixer (Xenyx 802, Behringer, Willich, Germany) merges the left and right channel receiver for stereo output. The Bluetooth transmitter (ABC01F, Aluratek, Irvine, CA, USA) sends the stereo audio signal to the audio eyeglasses by using the wireless connection.

**Figure 11:**
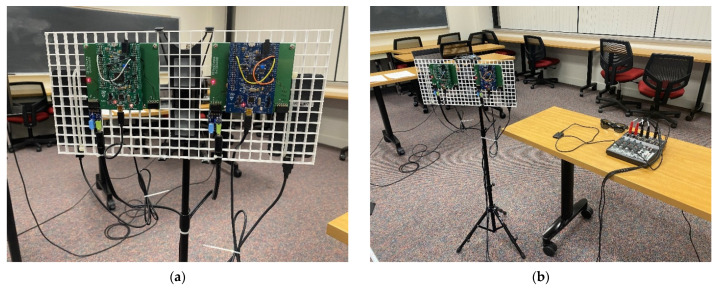
Actual eyeglass receiver device realization: (**a**) Dual channel receiver hardware; (**b**) Overall eyeglass receiver device hardware.

Figure 11 presents the actual eyeglass receiver device realization. The left and right channel receiver are located at the eggcrate panel for acoustic transparency. The distance between the left and right microphone is approximately specified as 19 cm according to the dimension of head and torso simulators [40,41,42]. The channel receivers are positioned at the speaker level by using the tripod and connected to the analog mixer for audio joining for stereo output. The Bluetooth transmitter is utilized for the connection between the stereo signal and audio eyeglasses. The eyeglass receiver device realized in this study is applied for the limited environment to investigate the directional localization. The static user wears the eyeglass receiver device to figure out the sound source direction which is controlled by the hidden person. Therefore, the tripod with channel receivers stays in the blind condition to stimulate the navigation situations for the visually impaired.

The embedded system for the channel receiver requires the STM32F4 series processor programming in the 32-bit Arm Cortex M4 architecture. The program controls the data acquiring, processing, and releasing in real time with tight schedule budget. Based on the sampling rate, the single data from the microphone is processed seamlessly for the DAC before the new data arrives. The IMP34DT05TR digital microphone in the channel receiver provides the data in pulse density modulation (PDM) which represents analog signal amplitude by using the relative density of the continuous pulses. However, the processing for the frequency shift and LPF require the direct quantization known as pulse coded modulation (PCM). The integrated firmware package in STM32F4 processor supports the library dedicated for the conversion from PDM to PCM. The approach and code structure in the channel receiver partially adopted the GitHub open-source programming [43] to the I^2^S audio output based on the PDM microphone.

**Figure 12:**
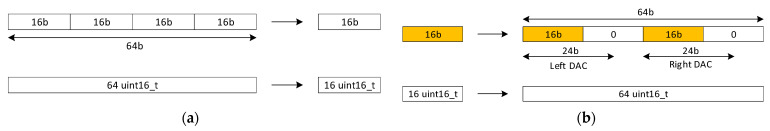
Data flow control for the receiver realization: (**a**) PDM to PCM conversion; (**b**) DAC insertion.

The conversion involves the filter and decimation process; hence, the data are compressed for reduced size in PCM form. In order to maintain the streaming in synchronized ADC and DAC system, the data in and out of the receiver process preserve the identical size to prevent the overflow and underflow on the information queue. Note that the channel receiver in Figure 9 shares the clocks for digital microphone and DAC for synchronization. Figure 12 demonstrates the data flow of the PDM to PCM conversion and DAC processing. For single 16-bit data, the conversion needs the 64-bit pulses from the digital microphone as shown in Figure 12a. The conversion function is designed to accept the 64-integer input for 16-integer output with 16-bit size integer. Observe that the decimation rate is 4 to 1.

In the DAC process, the reverse insertion procedure is necessary for consistent data rate. The single 16-bit is expanded for the 64-bit data frame for DAC, as shown in Figure 12b. The DAC receives the data in I^2^S Philips standard [44] with 24-bit data package on 32-bit frame as left alignment. The 16-bit data duplicated for left/right channel with the arrangement generates the 64-bit data frame for the single DAC instance. Similar to the PDM to PCM conversion, 16-integer input is expanded for the 64-integer output in the insertion procedure in Figure 12b. Therefore, the 16 integers from PDM microphone generate the 64 integers for DAC over the conversion and insertion. Note that the expansion rate is also 1 to 4.

**Figure 13:**
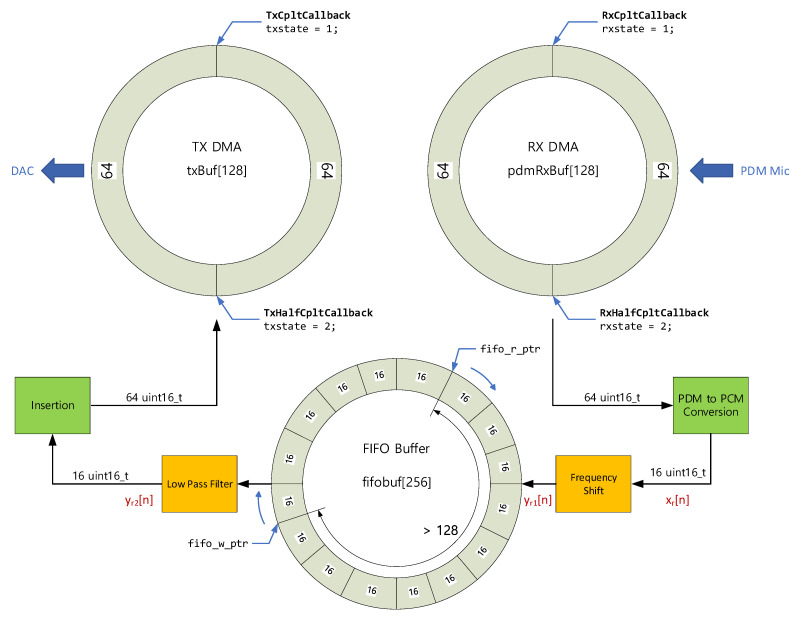
Software structure for the receiver realization.

The data management in conversion and insertion assures the flow between the digital microphone and DAC once the schedule is deployed in deterministic manner. In general, the acquisition and release of real-time data are performed by the interrupt and direct memory access (DMA) on embedded programming environment. Hence, certain statistical scheduling is observed in low time budget situations which may create the race condition. To prevent the unpredictable outcome, first-in first-out (FIFO) buffer is placed between the conversion and insertion as shown in Figure 13. Observe that all buffers are operated in circular fashion for continuous and real-time data processing. The 64-integer data are obtained from the digital microphone, decimated/processed by the receiver algorithms, and expanded/released to the DAC in the 64-integer data frame. The receiver and transmitter DMA fire the proper interrupt and assign the processor state for timing and buffer management. After the PDM to PCM conversion stage, the 16-integer data are written in the 256-integer FIFO buffer. Once the write pointer is greater than the read pointer in 128-integer, the system allows for the release of the 16-integer data toward DAC. The FIFO buffer provides the consistent access order to maintain the signal integrity by sacrificing the minor latency, which is 2.7 ms maximum for 48 kHz sampling rate.

The frequency shift and LPF are located before and after the FIFO buffer, respectively, as shown in Figure 13. For shifting operation, the simple multiplication in Equation (1) presents the frequency inversion which flips the frequencies between the highest and zero. Note that the DTFT indicates the discrete-time Fourier transform. The operation sampling rate for the development board is derived as 46.875 kHz for 48 kHz assignment due to the integer division from processor base clock. The mismatch in sampling rate should be considered in the transmitter algorithm for proper audible sound. The frequency shift in transmitter modulates the audio sound to 23.4375 kHz (= 46.875/2 kHz) in center frequency in order to equalize between the transmitter and receiver modulation.

(1) $$y_{r1}\left[n\right]={\left(-1\right)}^{n}x_{r}\left[n\right]\overset{\mathrm{DTFT}}{↔}Y_{r1}\left(e^{j\omega }\right)=X_{r}\left(e^{j\left(\omega -\pi \right)}\right)$$

The LPF in the receiver is realized by the finite impulse response (FIR) type with length of 21. Equation (2) demonstrates the FIR filter with impulse response $h_{r}\left[n\right]$ in time domain and with frequency response $H_{r}\left(e^{j\omega }\right)$ in corresponding frequency domain. Note that the N is the filter length as 21 and $\omega_{c}$ is the cutoff frequency which corresponds to the $f_{c}$ as 3.4 kHz. The STM32F4 processor contains the dedicated architecture for floating-point processing as FPU for single precision (SP) floating-point number. The FPU performs the SP multiplication and addition in one processor cycle individually [45]. Usually, the FIR filter coefficients are small decimal numbers, and the fixed-point number conversion is not necessary in this realization due to the FPU.

(2) $$y_{r2}\left[n\right]=\overset{N-1}{\underset{k=0}{\sum }}y_{r1}\left[n-k\right]h_{r}\left[k\right]\overset{\mathrm{DTFT}}{↔}Y_{r2}\left(e^{j\omega }\right)=Y_{r1}\left(e^{j\omega }\right)H_{r}\left(e^{j\omega }\right)$$

**Figure 14:**
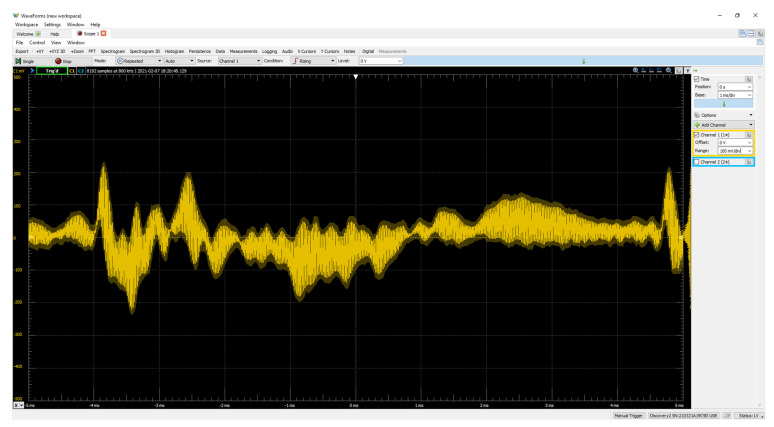
Actual output signal from the receiver in time domain.

Figure 14 illustrates the receiver output measured by oscilloscope in time domain. While the transmitter device broadcasts the ultrasonic sound with voice message, the receiver device accepts the ultrasonic sound and performs the receiver processing for the output. The speaker emits the clear voice message propagated from the transmitter device. The ambient sound is properly blocked by the receiver processing including the frequency shift and LPF. Therefore, the receiver device selectively delivers the transmitter signal for the user.

Two channel receivers are combined in the receiver device for binaural sound localization on human auditory system. The individual channel receiver presents the consistently low latency due to the embedded processing on dedicated processor. The perceived time difference between channels contributes to finding the transmitter direction based on the sound heard from the receiver device. In addition, the inter-channel magnitude variation derived from the distances expects to provide the complementary information for transmitter localization because of the inverse square law on acoustic signal propagation. Observe that the receiver and transmitter device in this study promotes the human sound localization capability for the ultrasonic frequency sound.

#### 3.1.2. Transmitter Device

The transmitter device plays the pre-recorded message in ultrasonic frequency range; therefore, the real-time processing is not required since the message is pre-determined. The transmitter is usually placed at the specific location to deliver the destination name or safety information. The transmitter device is realized by the personal computer with audio file which is recorded and preprocessed based on the transmitter algorithm. The USB audio interface converts the audio file into the analog signal by utilizing the high-fidelity audio-grade DAC. The analog signal is released into the air with proper amplifier and speaker. The frequency response of the audio hardware should be operated on the ultrasonic frequency range to deliver the message for the receiver device.

**Figure 15:**
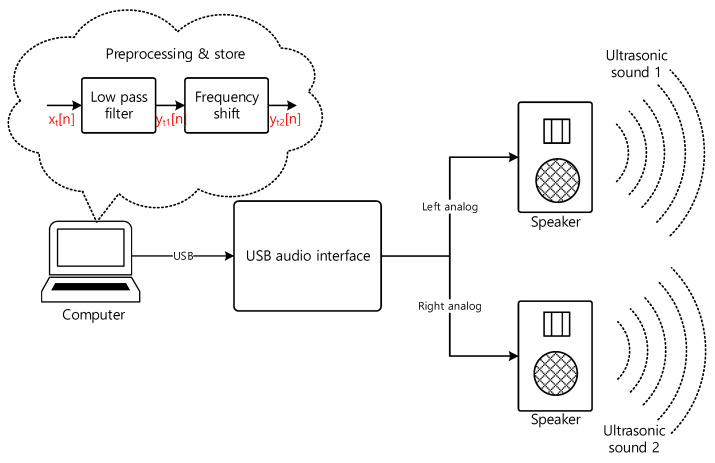
Transmitter device realization.

Figure 15 demonstrates the transmitter implementation. The audio message is recorded by the audio software (Audacity; open-source program) with microphone (Procaster, Rode, Sydney, Australia) and audio interface (M4, MOTU, Cambridge, MA, USA) in 96 kHz sampling rate at acoustically untreated room. The LPF and frequency shift are realized by MATLAB program with 101-length FIR filter and sinusoid multiplication, respectively. The audio file in wave format is produced by MATLAB from the filtered and shifted audio data. The USB audio interface (SSL2+, Solid State Logic, Begbroke Oxfordshire, England) plays the audio file for analog audio output. The powered speakers (A5X, ADAM Audio, Berlin, Germany) are connected from the audio interface via balanced analog wire for delivering the sound over the air. Note that, in highest sampling rate, the interface and speaker can be operated on the frequency up to the 80 kHz and 50 kHz, correspondingly.

**Figure 16:**
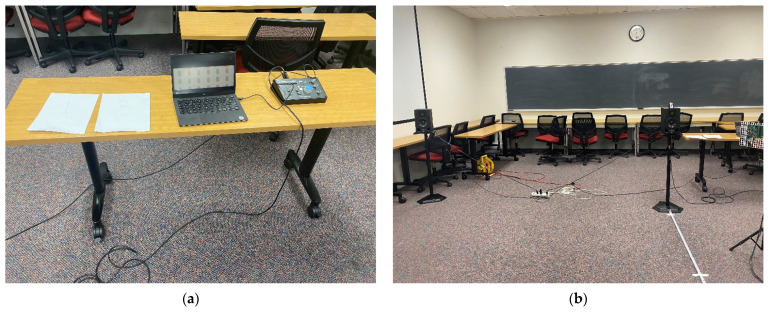
Actual transmitter device realization: (**a**) Computer and USB audio interface; (**b**) Two speakers with 3 m apart.

Figure 16 shows the actual transmitter device realization. The notebook computer in Figure 16a contains the audio file for transmitter message and the audio interface is connected to the notebook via USB connection. The speakers in Figure 16b are linked to the interface over the balanced analog connection. The audio file includes the stereo channel voice signal for left and right speaker; hence, two messages are propagated to the receiver device simultaneously at distinct locations. The speakers in Figure 16b exhibit the discrete locations with 3 m apart for spatial audio propagation. In the experiments, the users are requested to recognize the direction of the sound source speaker for the specific message. The other speaker continuously interrupts the user’s hearing process in order to investigate the multi-source localization performance, which is essential in conventional situations.

The LPF and frequency shift in transmitter device provide the sound message in ultrasonic frequency range. Equation (3) demonstrates the FIR LPF with impulse response $h_{t}\left[n\right]$ in time domain and with frequency response $H_{t}\left(e^{j\omega }\right)$ in corresponding frequency domain. The LPF cutoff frequency $\omega_{c}$ is derived from the receiver device sampling rate $f_{rs}$ (46.875 kHz) to maintain the transmitter output frequency above 20 kHz. Note that the FIR LPF length is 101 which is the N value in the filter.

(3) $$y_{t1}\left[n\right]=\overset{N-1}{\underset{k=0}{\sum }}x_{t}\left[n-k\right]h_{t}\left[k\right]\overset{\mathrm{DTFT}}{↔}Y_{t1}\left(e^{j\omega }\right)=X_{t}\left(e^{j\omega }\right)H_{t}\left(e^{j\omega }\right)$$

$$\omega_{c}=2\pi f_{c}\;@\;f_{c}=3.4\;\mathrm{kHz}$$

The frequency shift is also related to the sampling rate from receiver device as $f_{rs}$. Equation (4) shows the frequency shift by using the sinusoid multiplication with shift amount $\omega_{m}$. The proper voice signal from the receiver device requires the equalization between the frequency shifts in receiver and transmitter device. The simple realization of receiver frequency shift in Equation (1) is feasible due to the dedicated parameter value in Equation (4). Observe that the receiver algorithm should be executed in real time with low and consistent latency.

(4) $$y_{t2}\left[n\right]=2\cos\left(\omega_{m}n\right)y_{t1}\left[n\right]\overset{\mathrm{DTFT}}{↔}Y_{t2}\left(e^{j\omega }\right)=Y_{t1}\left(e^{j\left(\omega +\omega_{m}\right)}\right)+Y_{t1}\left(e^{j\left(\omega -\omega_{m}\right)}\right)$$

$$\omega_{m}=2\pi f_{m}=2\pi \frac{f_{rs}}{2}=2\pi \frac{46.875k}{2}$$

**Figure 17:**
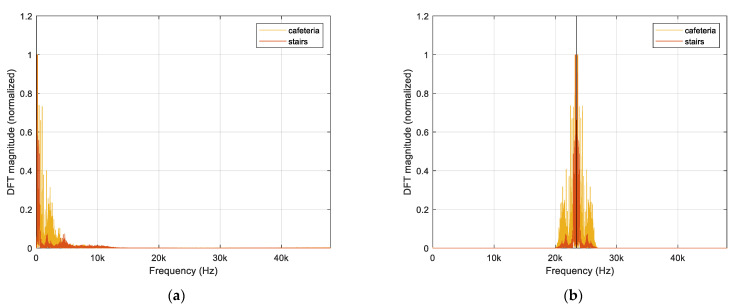
Normalized frequency distribution (based on discrete Fourier transform) of the signals $@\;f_{ts}=96\;\mathrm{kHz}$: (**a**) Sampled signal; (**b**) Band limited and frequency shifted signal $@\;f_{m}=46.875/2\;\mathrm{kHz}$ and $f_{c}=3.4\;\mathrm{kHz}$.

The LPF in Equation (3) and frequency shift in Equation (4) are implemented by MATLAB program with $f_{ts}$ (96 kHz) sampling rate for the voice signal. Two voice messages are “Cafeteria” and “Stairs” from female and male participant, respectively. Figure 17a illustrates the frequency distribution of the two messages. In frequency domain, the major power is allocated below 5 kHz; however, the minor power can be detected above 10 kHz as well. Figure 17b depicts the frequency distribution of the processed message for the transmitter device. With given $\omega_{c}$ and $\omega_{m}$ from Equations (3) and (4), practically the entire power is located above 20 kHz in the spectrum; therefore, the person cannot hear any sound from the transmitter device. Note that the black vertical line in Figure 17b specifies the $f_{m}$ value derived from $\omega_{m}$ in Equation (4).

The computer plays the bandlimited and frequency shifted messages for audio interface with dedicated powered speakers. The DAC rate of the audio interface is 96 kHz; therefore, the theoretically usable frequency is up to 48 kHz, which includes the frequency distribution of the message. The message from the transmitter device is inaudible but deliverable in power in ultrasonic frequency range. To protect the observer and participants auditory system, unprocessed initial messages are played for assessment of sound level by the meter (R8050, REED Instruments, Wilmington, NC, USA). The working area in the experiment room is maintained at the sound level under 70 dB which is below the Centers for Disease Control and Prevention (CDC) guideline. Note that the user should not be exposed to the more than 85 dB noise level for 2 h according to the CDC recommendation [46]. Table 1 shows the summary of parameters for transmitter and receiver device.

### 3.2. Scenarios

The experiments for USG system consist of two categories: human perception and acoustic performance. In the human perception, the users are requested to estimate the directions of transmitter device in blind condition. The acoustic performance experiment measures the actual time delay between the two receiver channels in various directions and distances. The experiments are arranged in the classroom with 3.0 m × 15.7 m × 7.7 m extent in height × length × width. One side of the classroom is covered by windows with vertical blinds and the other side contains a simple wall with two entrance doors. Blackboards are placed at the front and back of the room. The floor is overlayed by hard carpet and all desks and chairs are located at the edge of the room. Therefore, the experiments are executed in the acoustically untreated room, which creates the multipaths in sound propagation.

#### 3.2.1. Human Perception

The human subject study in the perception complies with USA. Federal Regulations and was approved by the Mercer University’s Institutional Review Board for Human Subject Research with approved protocol number H2109193. In addition, all participants and organizers fulfilled the guideline from CDC to prevent COVID-19 spread by wearing face masks and applying sanitization. Eight people participated in the experiment: 3 females and 5 males with ages ranging from 18 to 22 years old. The experiments established confidentiality by excluding any identification data except gender and age. Note that the participants can revoke or decline any or all of the experiment procedure by declaring discomfort.

**Figure 18:**
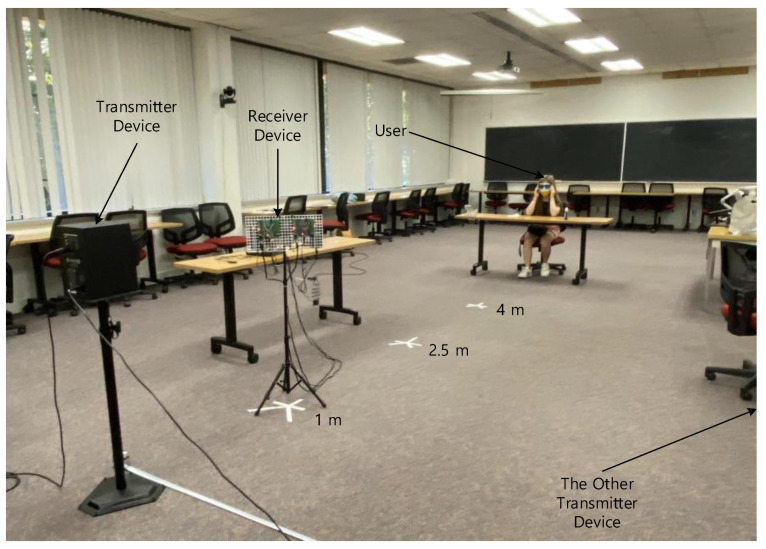
Human perception experiment configuration.

In the perception experiment configuration, the transmitter device is placed at the fixed location and the receiver device is moving along with the front radial line of the propagation as shown in Figure 18. The participant is seated at the other end of the classroom to determine the direction of transmitter device with respect to the receiver device. The participant remains blind by wearing the blindfold for whole procedure; therefore, the participant cannot recognize the receiver device movement. Observe that the receiver device and the user audio eyeglasses are connected by Bluetooth connection. The receiver device can be placed at 1 m, 2.5 m, and 4 m away from the transmitter device with 0,  ±45°,  and ±90° angles. The other transmitter device is located on the identical parallel line of the transmitter device, 3 m away, to distract from the user’s localization. One transmitter device emits the “Cafeteria” voice message and the other transmitter device transfers the “Stairs” message continuously.

#### 3.2.2. Acoustic Performance

**Figure 19:**
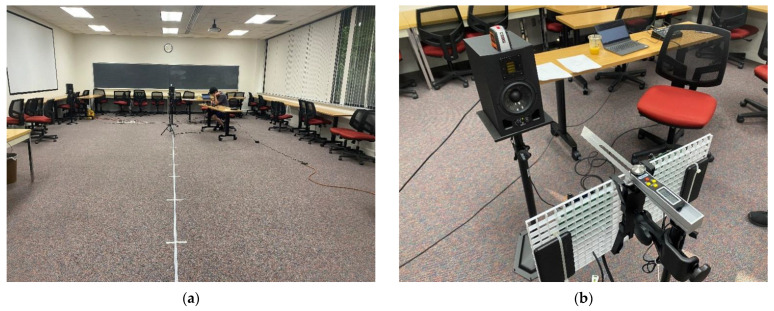
Acoustic performance experiment configuration: (**a**) Distance markers for receiver device; (**b**) Receiver device with digital protractor.

The experiment for acoustic performance measures the time difference between the receiver device channels. Overall configuration of the acoustic performance experiment is demonstrated in Figure 19. With single transmitter device, the receiver device changes the reception directions as 0°,  ±30°,  ±60°,  and ±90° and moves the reception distances from 0.5 m up to 7.0 m in 0.5 m resolution. The floor markers in Figure 19a indicate the placement points for receiver device. The perpendicular direction to the receiver line as shown in Figure 19b specifies the 0° evaluated by digital protractor (T-Bevel 828, General Tools and Instruments, Secaucus, NJ, USA) and the clockwise direction presents the positive angle. The positions and angles of the receiver are placed with naked eyes; hence, the measured performance contains the human estimation inaccuracy with probability.

The cross-correlation shown in Equation (5) estimates the displacement as well as similarity between two signals as left channel $x_{L}\left[n\right]$ and right channel $x_{R}\left[n\right]$. In the given system configuration, the left and right receiver channel accept the identical ultrasonic sound with time difference based on the receiving direction. Therefore, Equation (5) provides the distribution of arriving time difference between channels. Observe that the d in Equation (5) indicates the samples in digitized signal.

(5) $$r\left[d\right]=\overset{N}{\underset{n=-N}{\sum }}x_{L}\left[n\right]x_{R}\left[n+d\right]$$

The sample difference between channels is derived from the sample which represents the maximum absolute value in cross-correlation as shown in Equation (6).

(6) $$\tau =\arg\underset{d}{\max}\left|r\left[d\right]\right|$$

The receiver device output is recorded by the digital recorder (H1n, Zoom Corporation, Tokyo, Japan) directly connected to the analog mixer at Figure 10. The stereo audio signal is sampled at 96 kHz for approximately 10 s of recording.

## 4. Results

**Figure 20:**
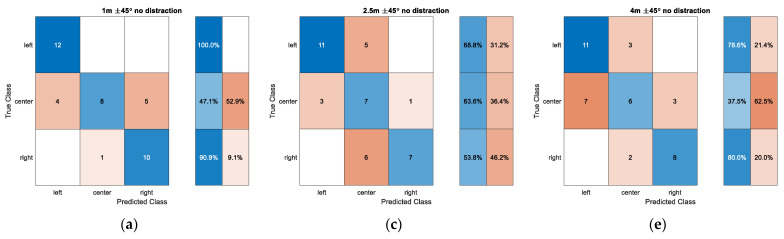

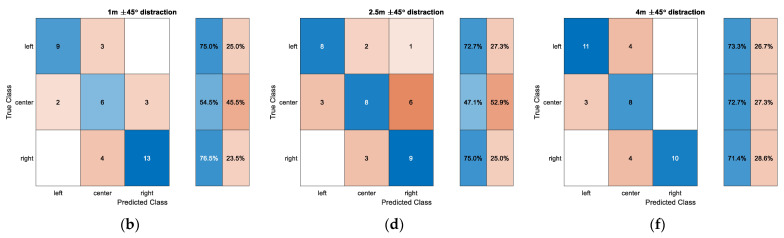
Confusion matrix for narrow angle situations: (**a**) 1 m and ±45° without distraction; (**b**) 1 m and ±45° with distraction; (**c**) 2.5 m and ±45° without distraction; (**d**) 2.5 m and ±45° with distraction; (**e**) 4 m and ±45° without distraction; (**f**) 4 m and ±45° with distraction.

At each distance, the observer asks the user for the direction of the sound source as left, center, or right. The user carefully hears the sound from the audio eyeglasses to determine the direction of the “Cafeteria” voice message while the user is distracted by the “Stairs” voice message in another direction. Figure 20 presents the confusion matrix for the narrow angle situation for individual distance. The left, center, and right direction indicates −45°, 0°, and 45°, respectively. In the confusion matrix, the true class specifies the actual direction of the sound source and the predicted class defines the user determined direction. The row summary on the right of the confusion matrix displays the number of correctly and incorrectly classified observations for each true class as percentages. Five tests are performed in each distance with the non-distraction and distraction situation for eight participants. The direction is given by the random number for uniform distribution.

In a short distance of 1 m, end directions are correctly predicted for the non-distraction situation with high likelihood; however, the center direction provides a low chance of reaching the true class. The distraction produces the lower performance for end directions as well as the center direction. The medium distance 2.5 m delivers the consistent performance around 60% accuracy for the non-distraction situation. The distraction condition exhibits the better predictions in end directions with marginal improvement; however, the center direction shows the predictions with high false population. The far distance of 4 m with non-distraction demonstrates around 80% accuracy for end directions but approximately 40% accuracy for the center direction. The distraction in 4 m provides the consistent accuracy for all directions of about 70%. Overall, the narrow angle situation derives the low performance in the center direction due to the shallow time difference with adjacent directions. The distraction barely contributes to the performance degradation except for the short distance situation.

**Figure 21:**
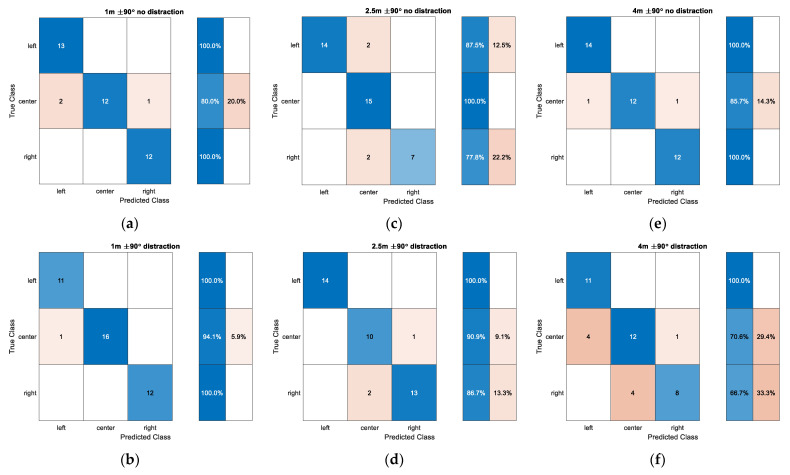
Confusion matrix for wide angle situations: (**a**) 1 m and ±90° without distraction; (**b**) 1 m and ±90° with distraction; (**c**) 2.5 m and ±90° without distraction; (**d**) 2.5 m and ±90° with distraction; (**e**) 4 m and ±90° without distraction; (**f**) 4 m and ±90° with distraction.

Figure 21 illustrates the confusion matrix for wide angle situations at individual distance. The left, center, and right direction indicates −90°, 0°, and 90°, respectively. The end directions in short distance are perfectly predicted for the non-distraction situation and the center direction still provides a good chance of estimating the true class. The distraction represents almost identical performance as the non-distraction case. The medium distance of 2.5 m shows the perfect prediction in the center direction and around 80% accuracy in the end directions. The distraction condition presents the similar performance pattern in all directions as the non-distraction situation with certain false prediction relocations. The far distance of 4 m with non-distraction demonstrates precise accuracy for end directions and approximately 85% accuracy for the center direction. The distraction in 4 m delivers the minor accuracy degradation in the center and right direction. In general, the wide-angle situation develops the improved performance in all directions with respect to the narrow angle counterparts due to the pronounced time difference between adjacent directions. The distraction barely contributes to the performance degradation except for the far distance situation.

**Figure 22:**
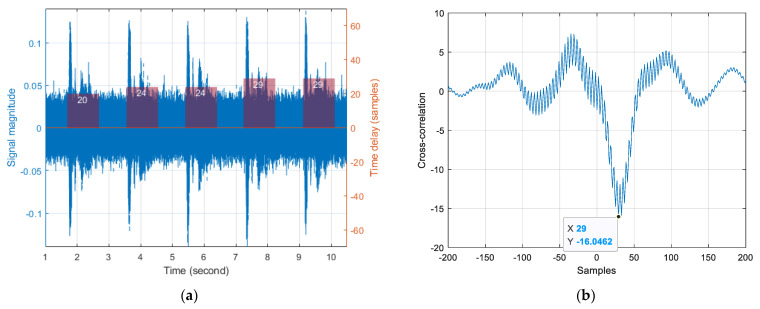
Signal and measured time delay distribution for 5.0 m and 30° configuration: (**a**) Signal magnitude in blue with left axis and measured time delay in transparent purple with right axis; (**b**) One output of cross-correlation.

Figure 22 shows the signal distribution and acoustic performance on the recorded audio from the USG system. The receiver device is located at 5.0 m away from the transmitter device with 30° direction. Figure 22a illustrates the signal distribution on the blue lines with left axis. Due to the long propagation distance, the noise in Figure 22a is noticeable and consistent. Note that the signal region is manually selected and marked by the purple bar span in the Figure 22a time axis. The selected signals with the stereo channel are individually contributed for computing the cross-correlation given by Equation (5) and presented by Figure 22b. The maximum absolute value in the cross-correlation indicates the inter-channel time delay, which is 29 samples in Figure 22b. The discrete measured time delays are written on the signal regions (transparent purple bars) in Figure 22a with the right axis. The ideal time delay for 30° is 27 samples according to Equation (7)

(7) $$\tau_{ideal}=\mathrm{round}\left(\frac{L\sin\left(\theta \right)}{C}f_{s}\right)$$

where L is inter-channel distance 19 cm, C is the sound speed 340.29 m/s, and $f_{s}$ is sampling frequency 96 kHz. The round function finds the nearest integer value.

Table 2 collects the averaged time delays between channels for various distances and directions. In order to maintain the far field provision, the distances are initiated from 0.5 m for measurement. The far field condition provides the plane wave propagation; therefore, the time delay is only a function of the receiver device angle. The ideal time delays are denoted in the last column of Table 2. The time delays in each row (same angle) demonstrate the fluctuation in distribution. The time delays from −60° to 60° show the consistent increment corresponding to the receiver device angle. However, the time delays in ±90° present the several inversion situations against the ±60° counterpart. The fluctuation and inversion circumstance are produced by the multipath propagations of the sound signal from the acoustically untreated environment.

**Figure 23:**
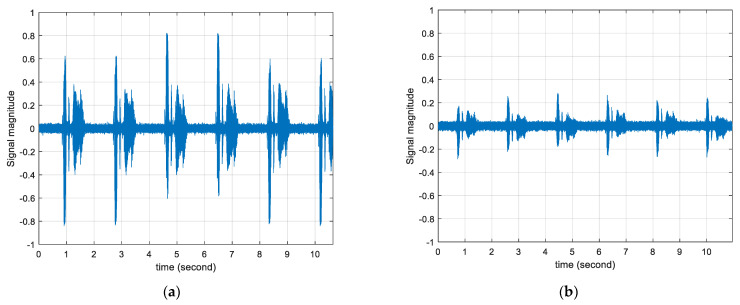
Signal distributions for 1.0 m distance: (**a**) Signal magnitude at 1.0 m with 60°; (**b**) Signal magnitude at 1.0 m with 90°.

Table 3 shows the means and standard deviations of measured time delays for individual angles of the receiver device. In an absolute sense, the means of the time delay relatively follow the ideal values except for the 60° and −90° cases. The inversion situation is observed between −60° and −90° mean time delay and the saturation condition occurs occurred between 60° and 90° mean time delay. The standard deviations are increasing toward the end directions in an asymmetric manner. Note that the ideal time delays are in a symmetric fashion but the mean time delays are distributed in a non-symmetric profile as well. The statistics for ±90° present high variations because of the low sensitivity to the sounds arriving at perpendicular directions. The digital microphone (IMP34DT05TR, STMicroelectronics, Geneva, Switzerland) used in the receiver device does not provide the polar pattern specification; however, conventional microphones with a single accept device depict the supercardioid or cardioid polar pattern which delivers the low signal gain from the side address situations. Figure 23 demonstrates the actual magnitude distributions at a 1 m distance with different angles. The magnitude in Figure 23a as 60° arrival presents a higher signal-to-noise ratio (SNR) than the magnitude in Figure 23b as 90° arrival.

The acoustic performance experiments provide the accurate time delays in center directions; however, the human perception experiments describe the improved estimations in end directions. Although the end directions produce the low SNR circumstance, the perpendicular directions deliver the elevated time delays which can easily be recognized by users. The statistical variation is wide in end directions; nevertheless, the time delays in the directions take longer to overcome the variation according to the Table 3 information. The experiments are performed in the acoustically untreated room which causes the multipath propagations. The fluctuation in Table 2 and asymmetricity in Table 3 of the time delay distribution are initiated from the non-ideal and realistic environment. The overall performance evaluations show the precise directional localization for narrow- and wide-angle situations even with the restricted system. Observe that the system does not provide any mobility for the localization. Therefore, the personalized wearable USG system is expected to develop enhanced performance for sound localization based on this analysis.

## 5. Conclusions

This study presents a novel sound guide system based on the ultrasonic sound to assist the indoor and outdoor navigation of the visually impaired. The transmitter devices are located at the points of interest to broadcast the frequency modulated voice message in ultrasonic sound. The receiver device is worn by the visually impaired person in the shape of eyeglasses so that they can receive the ultrasonic sound and identify the message via demodulation. The dual channel configuration of the receiver device provides the acoustical clue to the receiver device user for localizing the multiple transmitter positions by human localization capability. The transmitter device is realized by the computer, audio-interface, and speakers for playback of the processed voice messages. The receiver device is implemented by the conventional off-the-shelf products for real-time processing from a digital microphone to audio eyeglasses. In the medium size classroom, the experiments are designed to measure human perception as well as acoustic performance in terms of directions and inter-channel time delay, respectively. Due to the low microphone gain from side addressing, the time delay between the channels demonstrates high variance and high bias in the end directions. However, the perception experiment shows the further prediction accuracy in the end directions compared to the center direction outcomes. Although low statistical performance is observed in the end directions, the user can recognize the raised time delay in the end directions effortlessly. The overall performance evaluations show the precise directional localization for narrow- and wide-angle situations.

The ultrasonic sound guide system significantly improves the personal navigation for the visually impaired by removing the position ambiguity of the destination. With preliminary realization, the system proves the localization performance by human perception and acoustic characteristics. Based on the analysis in this study, several enhancements can be proposed in the realization of the optimized ultrasonic sound guide system. The all-in-one receiver device provides the head and neck mobility corresponding to the device direction. The simultaneous and instantaneous mobility is considerably dominant for sound localization since the binaural sound information is diversified from the movement. The personalized receiver device can adjust the distance between the channels for accurate estimation because the head and ear profile are applied for the distance. In addition, the acoustic training with the receiver device can develop the precise localization. The given receiver device in this article is implemented by the front-facing microphones which cause weak signal acceptance from the perpendicular directions. The angle modification and/or multiple microphones in the channel produce the high-fidelity sound from the wide angle. Future works may focus on the enhancement of the proposed system with a novel structure and shape, for instance, a single channel receiver, a neckband receiver device, etc. Additionally, future works will investigate the localization performance in various situations, indoors and outdoors, with dedicated configurations. The application of the proposed system can be extended beyond the sound guide system since the ultrasonic sound can be generated from wildlife and man-made systems. The system can monitor the locations of many animals such as bats, insects, and reptiles as well as leaking pipes. Future work will evaluate the system to validate further applications.

## 6. Patents

Resulting from the work reported in this manuscript, the USPTO patent application 17/396,694, filed 08/07/2021, under the title of “Ultrasonic sound guide system for the visually impaired” was written by Kevin Kim, Saea Kim, and Tae Choi.

## Figures and Tables

**Table 1:** Summary of parameters for transmitter and receiver device.

Block	Parameter	Transmitter Device	Receiver Device
ADC	Sampling frequency (Hz)	96 kHz	46.875 kHz
	Bit resolution (bits/sample)	16 bits/sample	16 bits/sample
Frequency shift	Shift amount (Hz)	23.4375 kHz	23.4375 kHz
LPF	Type/Length	FIR/101	FIR/21
	Cutoff frequency (Hz)	3.4 kHz	3.4 kHz
DAC	Sampling frequency (Hz)	96 kHz	46.875 kHz
	Bit resolution (bits/sample)	16 bits/sample	24 bits/sample

**Table 2:** Mean time delays between channels for specific distances and directions.

Dist. vs. Ang.	0.5 m	1.0 m	1.5 m	2.0 m	2.5 m	3.0 m	3.5 m	4.0 m	4.5 m	5.0 m	5.5 m	6.0 m	6.5 m	7.0 m	Ideal
90°	65	68	48	63	58	48	52	45	43	65	58	67	44	47	54
60°	63	64	53	61	50	60	46	35	59	45	58	59	54	44	46
30°	27	36	26	37	18	35	15	17	30	25	33	28	25	31	27
0°	0	0	−2	5	5	6	2	11	12	14	0	−4	−1	1	0
−30°	−25	−24	−25	−31	−31	−40	−31	−30	−24	−18	−40	−22	−34	−21	−27
−60°	−58	−43	−47	−53	−42	−59	−44	−60	−53	−40	−57	−40	−42	−49	−46
−90°	−58	−42	−55	−-50	−48	−47	−43	−51	−61	−58	−51	−53	−40	−30	−54

Unit: samples.

**Table 3:** Statistics for measured time delays for receiver device angles.

Stat.	90°	60°	30°	0°	−30°	−60°	−90°
Mean	54.92	53.61	27.49	3.62	−28.12	−49.11	−48.95
Stand. dev.	9.27	8.59	7.07	5.46	6.74	7.56	8.31
Ideal	53.60	46.42	26.80	0.00	−26.80	−46.42	−53.60
|Mean−Ideal|	1.32	7.19	0.69	3.62	1.32	2.69	4.65

Unit: samples.

## Data Availability

The acoustic data collected in the experiments for various distances and angles are available on request.

## Abbreviations

Listed by order of appearance in the text.

GPS	Global positioning system
3D	Three-dimensional
USG	Ultrasonic sound guide
ADC	Analog-to-digital converter
LPF	Low pass filter
DAC	Digital-to-analog converter
FPU	Floating-point unit
I^2^S	Inter-IC sound
MCK	Master clock
CK	Serial clock
WS	Word select clock
SD	Serial data
PDM	Pulse density modulation
PCM	Pulse coded modulation
DMA	Direct memory access
FIFO	First-in first-out
DTFT	Discrete-time Fourier transform
FIR	Finite impulse response
SP	Single precision
CDC	Centers for disease control and prevention
SNR	Signal-to-noise ratio
